# Investigating the efficacy of Endolift laser and Nanofat as a combination therapy for horizontal neck lines compared to Nanofat autologous alone

**DOI:** 10.1111/srt.13907

**Published:** 2024-09-02

**Authors:** Mohammad Ali Nilforoushzadeh, Shohreh Rafiee, Maryam Heidari‐Kharaji, Tannaz Fakhim, Niloufar Najar Nobari, Mohammadhasan Shahverdi, Zahra Lotfi, Sona Zare, Elham Torkamaniha, Shiva Alavi

**Affiliations:** ^1^ Skin and Stem Cell Research Center Tehran University of Medical Sciences Tehran Iran; ^2^ Jordan Dermatology and Hair Transplantation Center Tehran Iran; ^3^ Department of Veterinay Medicine and Surgery, University of Missouri, Columbia Missouri USA; ^4^ Department of Dermatology Rasool Akram Medical Complex Iran University of Medical Sciences Tehran Iran; ^5^ Laser Application in Medical Sciences Research Center Shahid Beheshti University of Medical Sciences Tehran Iran; ^6^ Stem Cell and Regenerative Medicine Center Sharif University of Technology Tehran Iran; ^7^ Department of Mechanical Engineering Sharif University of Technology Tehran Iran; ^8^ Department of Microbial Biotechnology Islamic Azad University Kish Branch Iran

**Keywords:** endolift laser, horizontal neck wrinkles, nanofat, treatment, wrinkles

## Abstract

**Background:**

The emergence of horizontal neck wrinkles is increasingly becoming a focal point for both cosmetic professionals and clients. Various treatment approaches must be considered to address this issue effectively, owing to its diverse underlying causes. The study explores the potential of utilizing the Endolift laser in conjunction with nanofat injection as a viable treatment option.

**Methods:**

Twenty patients with horizontal neck wrinkles involved in the study. Ten patients underwent treatment with a combination of Endolift laser and nanofat injection and 10 patients treated with nanofat injection alone. The participants were monitored for 6 months post‐treatment. Biometric measurements were utilized to assess outcomes, including changes in volume, depth, and area of the wrinkles, skin elasticity, as well as the diameter and density of the epidermis and dermis in the treated area. Skin improvement was evaluated by two independent dermatologists, who compared before and after photos in a blinded manner. Patient satisfaction levels were also documented.

**Results:**

The Visioface analysis showed a notable decrease in neck wrinkle depth and area in both groups. However, the group receiving the combination treatment of Endolift laser and nanofat exhibited a significantly greater improvement compared to the group treated with nanofat alone. Skin ultrasonography results demonstrated an increase in thickness and density of the dermis and epidermis in both groups. Particularly, the group treated with Endolift laser‐nanofat displayed significant enhancements in dermis and epidermis density and thickness when contrasted with the nanofat‐only group. Analysis with Cutometer revealed a marked enhancement in skin elasticity in the Endolift‐nanofat treated group in comparison to the nanofat‐only treated group. Furthermore, in the Endolift‐nanofat treated group, a substantial majority (90%) of patients exhibited improvement. Patient evaluations highlighted significant distinctions between the two groups, with 95% of patients in the Endolift‐nanofat treated group demonstrating enhancement.

**Conclusion:**

Both methods notably enhance horizontal neck wrinkles; nevertheless, the combination of endolift laser and nanofat seems to be more efficient for treating horizontal neck wrinkles.

## 1 INTRODUCTION

The aging of the neck is often one of the initial indicators of various age‐related changes. As more individuals desire a more youthful appearance, addressing neck aging has become a significant concern. This shift has led to a notable rise in approaches and ideas for improving the appearance of an aging neck. The aesthetic issues related to the neck encompass alterations in contour (e.g., platysmal bands, wrinkles, midneck hollowing, submental fat pads, or undefined jawlines) and changes linked to skin aging (e.g., neck wrinkles, loose skin, uneven texture, and hyperpigmentation).^1^, ^2^ Brandt and colleagues^3^ published a study detailing four specific categories of neck degeneration associated with aging. The neck skin becomes thinner with fewer sebaceous glands, making it prone to dryness due to reduced moisture. Additionally, the loss of collagen and connective tissue atrophy contribute to more noticeable changes in neck skin quality, often appearing before visible contour alterations.^4^ The aging of the skin on the neck is influenced by various factors, including both internal and external elements. Internal aging results from natural aging processes and genetic influences, while external factors like sun exposure, pollution, smoking, and diet also play a significant role. Furthermore, repetitive movements of the neck, such as twisting or tilting the head, can strain the neck and impact skin elasticity.^5^ The development of neck wrinkles is influenced by a range of factors, making it a multifaceted issue. By addressing external aging factors, it is possible to combat the aging process effectively. Individuals seeking assistance for neck lines are often younger and commonly adopt a forward‐leaning posture due to prolonged use of devices like mobile phones and computers. Consequently, neck wrinkles are increasingly recognized as a significant aspect of age‐related neck changes, not limited to older individuals.^6^ As awareness of neck aesthetics grows, researchers and healthcare professionals are giving more attention to changes in neck appearance. Autologous fat grafting has gained significant popularity in the field of tissue reconstruction and augmentation in the last decade. Various studies have observed its regenerative properties and positive effects on skin texture.^7^, ^8^, ^9^ Factors like hyperpigmentation, skin texture, and scar quality are crucial in determining the most suitable treatment and desired results. In this context, Tonnard and colleagues introduced a new technique involving “nanofat,” a refined form of lipids created through the mechanical emulsification of microfat, suggesting its potential applications in addressing skin conditions such as wrinkles and discolorations. Nanofat comprises tissue stromal vascular fraction (t‐SVF) and adipose‐derived stem cells (ASCs), giving ASCs valuable regenerative and rejuvenating qualities.^10^ Traditional approaches for wrinkles, like fillers, can be costly and temporary. Therefore, the long‐term regenerative properties of nanofat may offer considerable benefits for treating wrinkles. Numerous studies have highlighted the application of nanofat as a dermal filler for skin rejuvenation, enhancing texture, and treating scars.^11^, ^12^, ^13^, ^14^ The Endolifting technique, originally known as endolaser or endolift, involves utilizing a 1470 nm wavelength laser beam delivered through an optical fiber inserted into the subdermal tissue. This approach aims to decrease wrinkles and enhance skin tone by stimulating neocollagenesis. Due to its minimally invasive nature, fast recovery time, and minimal disruption to daily activities, it has gained popularity in recent years and is increasingly available in various locations offering cosmetic procedures.^15^, ^16^, ^17^, ^18^, ^19^, ^20^, ^21^, ^22^, ^23^

Given all of that, this study aimed to examine how combination therapy with nanofat and endolift laser impacts enhancing skin quality, in reducing neck wrinkles. Additionally, it aimed to discuss the safety, biometric assessment findings, and post‐treatment patient satisfaction.

## 2 METHODS

### 2.1 Patients

This research involved 20 patients with horizontal wrinkles on their neck, aged between 30 and 50 years. They were divided into two treatment groups: in one group, the horizontal neck wrinkles were treated with combination of Endolift laser and nanofat, (Endolift‐nanofat treated group), and in the other group, only the horizontal neck wrinkles were treated only with nanofat (nanofat only group). Patients with coagulation defects, pregnancy, local ulcers, autoimmune diseases, infectious diseases, cancer, or those undergoing any treatments were excluded. Treatment methods included Lidocaine 10.56% cream, local lidocaine HCI 2%, and epinephrine 1:1 000 000 infiltration, or sedation based on patient preference. Post‐treatment, all patients were prescribed empirical amoxicillin clavulanate 1 g three times daily for 7 days and oral cortisone for 5 days. The patients were thoroughly informed and given detailed instructions about the treatment.

### 2.2 Statement on ethics

All patients provided informed consent. Standard demographic information was collected using special questionnaires. Patients were given a thorough explanation of the study's design, purpose, and expected outcomes. All patients were assessed before and 3 months after completing the treatment sessions (IR.TUM.MEDICINE.REC1403.048‐Research ethics committee of medical school‐ Tehran University of Medical Sciences. Identification number for the trial registration: IRCT20200127046282N47).

### 2.3 Nanofat processing technique

#### 2.3.1 Donor site selection

Potential donor sites identified for fat harvesting include the lower abdomen and thighs.

#### 2.3.2 Prepare the lipoaspiration sites and fat harvesting

Patients should be marked while standing to accurately identify the areas with excess or undesired fat deposits. Once the skin is prepped and draped, a modified Klein's tumescence solution consisting of 500 mL of NaCl 0.9% solution, 1 mg/mL of adrenaline (1:10 000), and 25 mL of lidocaine (20 mg/mL) is slowly infiltrated into the skin using a specialized instrument. The infiltration is carried out through a 2 mm incision in the donor area using a “wet” technique. In this procedure, 120 cc of mixed fat with tumescent solution is manually harvested from the subcutaneous fat in a radial pattern resembling the spokes of a wheel. A Tonnard harvester with a 2.4 mm cannula and sharp 1 mm diameter holes is employed, collecting the fat mixture in a 20‐mL Luer Lock syringe.

During the processing and washing stage, the syringe containing the harvested material is vertically positioned to separate the layers. Adipose grafts settle in the middle, with a lipid layer on top, while the lower fluid is discarded. Expect a fat graft yield of 1.5 mL per 5 mL aspirate, resulting in around 40 cc of microfat. The donor area is massaged, excess fluid drained, incisions sutured, and a sterile dressing applied for compression. The liquid layer is removed, ensuring not to combine it with the grafts to prevent complications. Finally, a wash with Ringer's lactate solution is done to remove residual fluids and cells.

#### 2.3.3 Emulsification procedure

Following the decantation process, the purified microfat is filled into 20 cc syringes and mechanically emulsified by transferring the contents back and forth 30 times between two 20 cc syringes linked with a 2.4 mm Tulip connector, then repeating the process with a 1.4 mm Tulip connector, and finally with a 1.2 mm Tulip connector until the fat turns into a liquid state and obtains a whitish appearance.

#### 2.3.4 Nanofat procedure

Nanofat processing involves passing emulsified fat through a nanotransfer block equipped with double filters of 400 mm and 600 mm single‐use cartridge net before transferring it into a 20‐cc syringe. The nanofat is subsequently transferred into 1 cc Luer Lock syringes for injection.

### 2.4 Treatment protocol

#### 2.4.1 Endolift laser

The Endolift (LASEMAR 1500 machine from Eufoton s. r. L)‐ based subcision was programmed as follows: Power 3‐3.5 watt/Pulse T ON 25, T OFF 75/Fiber 400 micron, Number 200–250 J on each side of the neck., the total shots for whole neck 400–500 J. In the Endolift‐nanofat group, the 300‐micron fiber was smoothly inserted just below the skin of horizontal neck wrinkle surface, reaching into the superficial dermis and moving along the subdermal plane parallel to the skin. A quick and repeated back‐and‐forth movement of the needle was used to scrape the underside of the dermis. Subsequently, a side‐to‐side motion was applied to fully release fibrous tissues. This procedure was carried out once. Images of patients were captured using Visio‐face before treatment initiation and after treatment.

#### 2.4.2 Injection of nanofat

The neck skin is prepped with betadine and lidocaine injection. Nanofat is injected intradermally into the horizontal neck wrinkle treated with Endolift laser to create small papule‐like bumps across the skin using a cannula (gage 18). In the nanofat‐only group, the patients were injected with nanofat in the horizontal neck wrinkles. The patients were followed up 6 months after treatment.

### 2.5 Efficacy evaluation

#### 2.5.1 Biometric assessment

Six months post‐treatment, the patients' biometric characteristics were evaluated using Visioface 1000 D, Cutometer, both obtained from Courage + Khazaka Electronics in Cologne, Germany, and a skin ultrasound imaging system (TPM, Luneburg, Germany). Visioface was employed to analyze variations in volume, depth, and area of the wrinkles. Patients were required to position their face inside the cavity for image capture. Consistent head positioning, lighting, and camera distance were maintained across sessions to ensure comparability of photos from different visits. The device automatically analyzed pore changes and generated quantitative data. Cutometer evaluated skin elasticity using three parameters (R2, R5, R7), with values closer to one indicating higher expected skin elasticity. Ultrasonography was used to measure the diameter and density of the epidermis and dermis, where greater density signified higher collagen content in the dermis resulting in improved outcomes.

#### 2.5.2 Patient assessment

After the treatment, patients were asked to fill out a questionnaire to assess their satisfaction with the treatment outcomes and report any observed side effects. They were also requested to rate the treatment results using a 7‐point global assessment scale ranging from significant deterioration (−3 points) to significant improvement (+3 points).

#### 2.5.3 Physician assessment

Photos taken by visioface at the beginning and 6 months post‐treatment were reviewed by a dermatologist. The dermatologist, unaware of the study protocol, assessed the photos on a 7‐point scale: significant deterioration (−3), moderate deterioration (−2), minor deterioration (−1), no change (0), minor improvement (+1), moderate improvement (+2), and significant improvement (+3).

### 2.6 Statistical analysis

The data collected underwent analysis using the SPSS Statistics version 28 software. Repeated measures ANOVA will be employed to compare the average variations in quantitative parameters like skin thickness and skin density. Pairwise group comparisons will utilize the Sidak method. Assessing qualitative variables such as patient satisfaction will involve the Chi‐square test. Continuous data are presented as mean ± standard deviation (SD). Significance was set at a *p*‐value of ≤0.05.

## 3 RESULTS

### 3.1 Biometric measurement outcomes

**FIGURE 1:**
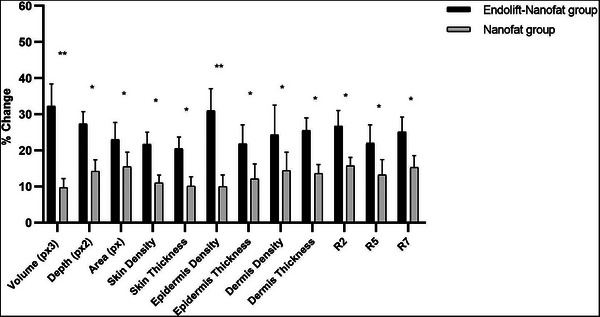
Comparing the mean biometric percentage change between the two groups.

The results of the biometric assessment before and 6 months after treatment are presented in Tables 1 and 2. The Visioface findings revealed a significant reduction in neck wrinkle depth and area in both groups. The percentage changes for depth and area were 27.38 ± 3.42 and 23.05 ± 4.73, respectively, in the Endolift laser‐nanofat group, and 14.28 ± 3.16 and 15.5 ± 4.03 in the nanofat‐only group (*p* < 0.05; see Figure 1, Tables 1 and 2). These results showed that Endolift laser‐nanofat was more effective than nanofat only for the horizontal neck treatment (*p* < 0.05). Additionally, skin ultrasonography data indicated increased thickness and density in the dermis and epidermis in both group (Figure 1; Tables 1 and 2). Significant improvements in epidermis and dermis density and thickness were observed in the group treated with Endolift laser‐nanofat in compare to group treated with nanofat only, with percentage changes in epidermis thickness and density being 21.83 ± 5.32 and 31.01 ± 6.11 in the Endolift laser‐nanofat treated group, and 12.13 ± 4.12 and 10.01 ± 3.21 in the nanofat‐only treated group, respectively. The dermis thickness and density increased by 25.51 ± 3.50 and 24.40 ± 8.19, respectively, in the Endolift laser‐nanofat treated group, whereas in the nanofat‐only treated group, the changes were 13.61 ± 2.52 and 14.40 ± 5.10 (*p* < 0.05; Figure 1, Tables 1 and 2). Significant differences were observed between the two groups' percentage of changes (*p* < 0.05), as indicated in Figure 1. Moreover, the cutometer results showed a significant improvement in skin elasticity in the Endolift laser‐nanofat treated group when compared to the group treated with nanofat only (*p* < 0.05) (Tables 1 and 2, Figure 1).

**TABLE 1:** Comparing biometric characteristics of the horizontal neck wrinkles in the Endolift nanofat groups, before and 6 months after the treatment.

	Measured values			
	Before	After	Percent change	*p*
**Visioface**				< 0.05
Wrinkle				
Volume (px^3^)	120.09 ± 15.17	70.66 ± 10.07	32.25 ± 6.16	
Depth (px^2^)	10.96 ± 3.57	7.08 ± 3.86	27.38 ± 3.42	
Area (px)	13.45 ± 5.17	9.23 ± 3.19	23.05 ± 4.73	
Skin ultrasonography				
Skin density	52.00 ± 4.31	71.21 ± 8.56	21.71 ± 3.36	
Skin thickness	1162.12 ± 100.02	1416.26 ± 106.87	20.52 ± 3.21	
Epidermis density	84.208 ± 10.716	131.09 ± 9.52	31.01 ± 6.11	
Epidermis thickness	95.40 ± 11.81	129.23 ± 12.35	21.83 ± 5.32	< 0.05
Dermis density	46.40 ± 3.58	65.25 ± 10.64	24.40 ± 8.19	
Dermis thickness	1069.25 ± 50.17	1341.80 ± 90.02	25.51 ± 3.50	
Elastisity^a^				
R2	0.75 ± 0.10	0.98 ± 0.08	26.79 ± 4.28	
R5	0.50 ± 0.11	0.69 ± 0.10	22.03 ± 5.08	< 0.05
R7	0.35 ± 0.045	0.49 ± 0.05	25.13 ± 4.16	

The data was shown as mean ± SD. The *p* < 0.05 was considered as statistically significant in all the tests.

^a^ Elasticity of the skin measured by Cutometer (mm/time).

**TABLE 2:** Comparing biometric characteristics of the horizontal neck wrinkles in the nanofat‐only groups, before and 6 months after the treatment.

	Measured values			
	Before	After	Percent change	*p*
**Visioface** Wrinkle				
Volume (px^3^)	109.39 ± 20.17	95.66 ± 21.27	9.78 ± 2.46	< 0.05
Depth (px^2^)	10.0 ± 2.7	8.0 ± 2.16	14.28 ± 3.16	
Area (px)	13.05 ± 3.17	10.13 ± 3.19	15.5 ± 4.03	
Skin ultrasonography				
Skin density	42.10 ± 4.31	50.21 ± 8.56	11.01 ± 2.16	< 0.05
Skin thickness	1132.02 ± 90.02	1256.26 ± 89.0	10.2 ± 2.51	
Epidermis density	94.08 ± 11.06	109.09 ± 10.12	10.01 ± 3.21	
Epidermis thickness	96.20 ± 11.81	115.23 ± 9.35	12.13 ± 4.12	
Dermis density	43.10 ± 6.58	54.05 ± 6.64	14.40 ± 5.10	
Dermis thickness	1018.15 ± 43.17	1241.60 ± 87.02	13.61 ± 2.52	
Elastisity^a^				
R2	0.70 ± 0.10	0.88 ± 0.08	15.78 ± 2.28	< 0.05
R5	0.47 ± 0.11	0.57 ± 0.10	13.23 ± 4.23	
R7	0.39 ± 0.015	0.49 ± 0.05	15.33 ± 3.26	

The data was shown as mean ± SD. The *p* < 0.05 was considered as statistically significant in all the tests.

^a^ Elasticity of the skin measured by Cutometer (mm/time).

### 3.2 Physician assessment

**FIGURE 2:**
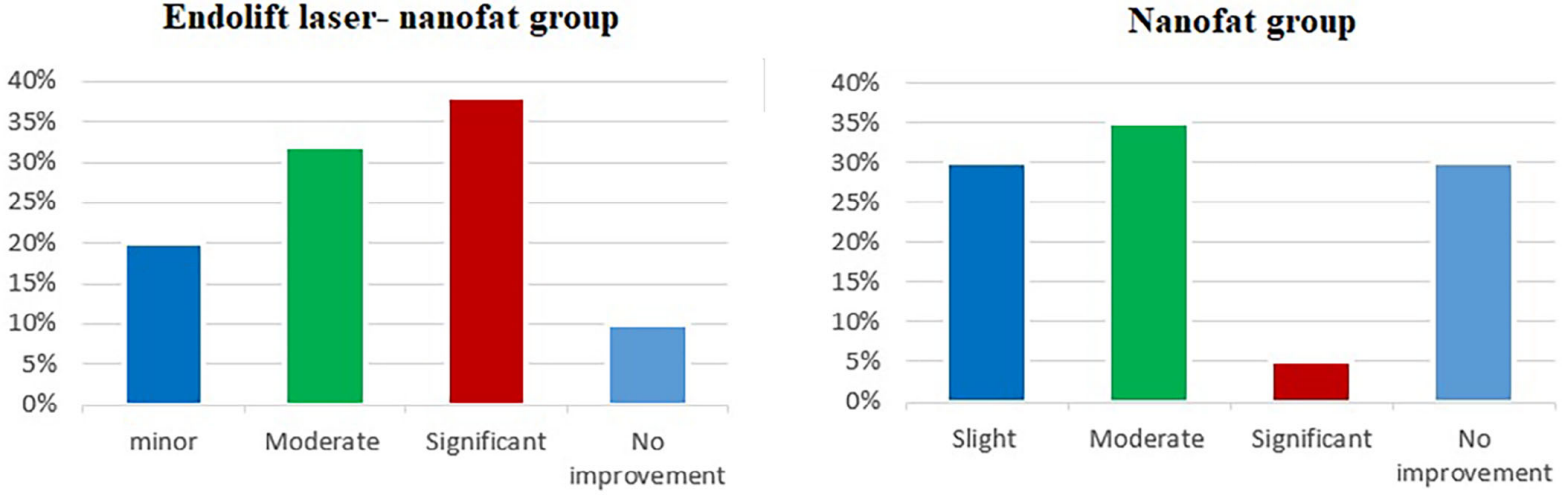
The pie chart displays the assessment of physician regarding the treatment.

The patient photographs were analysis by two blinded dermatologists and the results indicate significant differences between the two groups, as shown in Figure 2. In Endolift laser‐nanofat treated group, a significant percentage (90%) of patients showed improvement, with 20% having minor improvement, 32% moderate improvement, and 38% significant improvement. Only 10% showed no improvement, and none experienced a decline (Figure 2). Conversely, in nanofat‐only group, the majority of patients (70%) demonstrated improvement, with 30% showing minor improvement, 35% moderate improvement, and 5% significant improvement. Additionally, 30% did not show improvement, and none displayed a decline, as illustrated in Figure 2.

### 3.3 Patient evaluation

**FIGURE 3:**
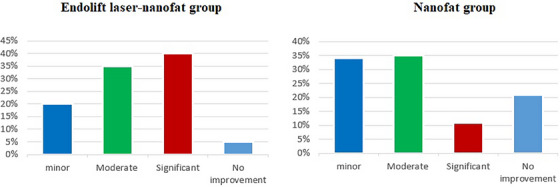
Pie chart displaying patients' satisfaction of the treatment.

The majority of patients were content with the treatment results. Evaluations from patients revealed significant differences between the two groups. In Endolift laser‐nanofat treated group, 95% of patients displayed enhancement: 20% exhibited minor improvement, 35% showed moderate improvement, and 40% demonstrated significant progress. Only 5% did not improve, with no cases of deterioration. On the other hand, in the nanofat‐treated group, 79% of patients saw improvement, with 34% reporting minor improvement, 35% moderate improvement, and 11% significant improvement. Furthermore, 21% did not improve, and there were no cases of deterioration. Figure 3 illustrates these findings.

## 4 DISCUSSION

Various surgical and nonsurgical techniques, either used independently or in combination, are available to confront neck aging and achieve long‐lasting neck rejuvenation results. Several cosmetic procedures, such as local skin tissue fillers and laser treatments, are available to target neck wrinkles effectively.^6^

The Endolift laser technique now offers a dependable alternative for 80% of patients compared to traditional methods, providing increased comfort for both patients and surgeons. This laser therapy improves wrinkles through two mechanisms: physical subcision like action by the fiber beneath the skin, and the stimulation of collagen and elastin production by the diode laser. Endolift is a noninvasive method without any side effects. The effect of Endolift laser on wrinkles and acne scar treatment was evaluated in several studies.^17^, ^21^, ^24^

In this research study, we observed positive outcomes when treating horizontal neck wrinkles using a combination of Endolift laser and nanofat injection. Analysis of skin ultrasound results indicated a significant enhancement in epidermal and dermal density and thickness in the group receiving Endolift laser‐nanofat treatment compared to the group treated with nanofat alone, 6 months after treatment (*p* < 0.05). Cutometer results also demonstrated noteworthy improvements in skin elasticity in the Endolift laser‐nanofat treatment group compared to the nanofat‐only group (*p* < 0.05). In addition, the physician assessment during the 6‐month follow‐up period, showing that in Endolift laser‐nanofat treated group, a significant percentage (90%) of patients showed improvement. Evaluation of patient satisfaction revealed a high satisfactory rate (95%) based on the last follow‐up data of all subjects in Endolift laser‐nanofat treated group.

Improving skin quality can be effectively achieved through various methods, but subcutaneous nanofat injections have emerged as a highly efficient technique for facial rejuvenation. This approach stands out for its ability to alter dermis patterns. Nanofat grafting, a regenerative procedure using the body's own materials, can be done on an outpatient basis. Clinical trials have confirmed the safety of this method, demonstrating seamless integration of nanofat with host tissues and minimal side effects.^25^

Nanofat includes stromal vascular fraction (SVF) and adipose‐derived stem cells (ASCs). The ASC differentiation process leads to a significant production of type I collagen, along with lesser amounts of type V and type VI collagen and proteins. This regeneration of fibroblasts, increased secretion of cell matrix, and repair of dermal breaks contribute to the reconstruction and revitalization of the skin structure, ultimately enhancing the appearance of wrinkles.^12^ ASCs are known for their vital role in regenerative technology. Typically placed just below the dermis, these grafts help restore lost tissue volume and stimulate collagen production by stretching dermal fibroblasts, enhancing skin texture and thickness over time.^26^

Research on nanofat indicates a boost in skin elasticity, possibly due to enhanced production of collagen and elastin, as well as restructuring facilitated by adipocyte stem cells disrupted during emulsification.^10^

Tenna and colleagues published a study indicating that application of nanofat with PRP (platelet‐rich plasma), either alone or combined with a fractional CO_2_ laser, demonstrated enhanced improvement in atrophic facial scars among 30 patients with skin types ranging from II to IV.^27^ Previous clinical studies have demonstrated that adipose tissue has regenerative properties in the dermis and subcutaneous tissue.^11^, ^28^, ^29^

Our findings support the use of a combined approach, which is more effective method for treating horizontal neck wrinkles to achieve optimal results compared to nanofat alone. Nanofat tissue has a very homogeneous structure. After Endolift subcision and nanofat injection under the horizontal wrinkles of the neck, it stimulates the connective tissue along with the Endolift fibers, which can open up fibrosis in the intervention area and promote tissue regeneration by receiving mesenchymal stem cells derived from nanofat tissue. This semi‐invasive combined therapy can serve as a semi‐permanent alternative to invasive plastic surgeries for lifting and tightening the neck skin.

## CONFLICT OF INTEREST STATEMENT

The authors declare no conflicts of interest.

## Data Availability

The data that support the findings of this study are available from the corresponding author upon reasonable request.
